# Genome-wide characterization of the *Rab* gene family in *Gossypium* by comparative analysis

**DOI:** 10.1186/s40529-017-0181-y

**Published:** 2017-06-02

**Authors:** Peng Li, Wangzhen Guo

**Affiliations:** State Key Laboratory of Crop Genetics and Germplasm Enhancement, Nanjing Agricultural University, Nanjing, 210095 Jiangsu Province People’s Republic of China

**Keywords:** *Rab* gene family, Genome-wide identification, Gene duplication, Phylogenetic relationship, Gene structure, Expression pattern

## Abstract

**Background:**

*Rab* protein family is the largest subfamily of small G protein family. As one of the most important families in plant, *Rab* family plays an important role in the process of plant growth and development. So far, the identification of 57 members of the *Rab* family in *Arabidopsis* has been completed. In cotton, the relevant family has not been reported.

**Results:**

Here, we identified 87, 169, 136, 80 *Rabs* in the four sequenced cotton species, *G. raimondii* (D_5_), *G. hirsutum* acc. TM-1 (AD_1_), *G. barbadense* acc. 3-79 (AD_2_) and *G. arboreum* (A_2_), respectively. Biological information analysis showed that the number of amino acid is 200–300 aa among *Rab* family members in *G. raimondii* and the protein molecular weight is between 20 and 30 kDa, which is consistent with the characterization of the *Rab* protein itself. 87 *GrRabs* in *G. raimondii* are divided into eight groups. In each group, intron numbers and subcellular localization of *Rab* protein are basically the same. We mapped the distribution of *GrRab* genes on 13 chromosomes of *G. raimondii* except two genes. Among the 87 *GrRabs* in *G. raimondii*, we identified 60 pairs of *GrRabs* formed in whole genome duplication. Among all the gene pairs, the *Ka/Ks* values were less than 1. This indicates that it is the results of the purification selection and will help maintain the conservation of gene in structure and function. Further, 4 of the 87 *GrRabs* showed tandem duplication. They were *GrRabA2a* vs *GrRabD1a* and *GrRabA2h* vs *GrRabD1b* respectively. Expression patterns analysis of 169 *GhRabs* in *G. hirsutum* acc. TM-1 indicates that most *Rab* family members play a certain role in different tissues/organs and different growth stages of cotton, implying their potential function in the polar growth of pollen tube, root hair and fiber cell, as well as improving stress and disease tolerance.

**Conclusion:**

The systematic investigation of *Rab* genes in cotton will lay a foundation for understanding the functional roles of different *Rab* members in the polar growth and stress tolerance.

**Electronic supplementary material:**

The online version of this article (doi:10.1186/s40529-017-0181-y) contains supplementary material, which is available to authorized users.

## Background

As the most important cash crop in the world, cotton provides a very important natural fiber for human beings. Therefore, we tried to explore the important genes related to abiotic stress, biotic stress and fiber development from the whole genome of cotton, hoping to improve cotton quality and yield. *Rab* protein family in cotton is a very large family. According to previous reports, *Rab* family is not only closely related to plant growth and development, but also plays an important role in stress and disease resistance.

The world’s first *Rab* gene (*YPT1*) was discovered in yeast in 1983 (Gallwitz et al. 1983). Four years later, Salminen and Novick demonstrated that *Ras* (*Sec4*) is involved in vesicle trafficking (Salminen and Novick 1987). In the same year, Tavitian and his colleagues cloned the homologous gene similar to the *SEC4/YPT* gene for the first time through cDNA library in rat brain and named ras-like in rat brain (*Rab*) (Martinez and Goud 1998). In *Arabidopsis thaliana*, 57 Rab proteins were found and divided into *RabA* to *RabH* (Hill and Sylvester 2007). *RabA* to *RabH* in *A. thaliana* correspond to *Rab11*, *Rab2*, *Rab1*, *Rab18*, *Rab8*, *Rab5*, *Rab7* and *Rab6* in animal, indicating that *Rab* family has further differentiation in plant (Pereira-Leal and Seabra 2001; Brighouse et al. 2010).

*Rab* protein family members have about 200 amino acids. Their sequences are conservative with high sequence similarity. All *Rab* proteins have five typical conserved domains, including four guanine nucleotide binding domains (G1, G3, G4 and G5) and an effector binding domain (G2) (Takai et al. 2001; Agarwal et al. 2009). Four domains of G1, G3, G4 and G5 participate in the binding and hydrolysis of nucleotides. Among them, G1 is the binding site of phosphate or Mg^2+^, G4 and G5 are key sites involved in GTP-GDP binding and hydrolysis (Stenmark and Olkkonen 2001). Mutations in the important amino acid sites of these conserved domains will cause *Rab* proteins to produce some constitutive inhibiting or activating mutant proteins. Normally, the amino acid sequence at the C end of the *Rab* protein is highly variable, but it ends with two conserved cysteine residues (CC) ultimately. These two highly conserved cysteine residues play an important role in membrane localization and protein function (Rutherford and Moore 2002). Although five G domains of *Rab* protein is discrete distribution in amino acid sequence, they are close to each other to form a special catalytic domain in the three-dimensional conformation of proteins, so as to better exercise the function of a protein (Rutherford and Moore 2002). The main function of the *Rab* protein family member is responsible for intracellular protein transport and they are essential regulator of vesicle trafficking way (Novick and Zerial 1997; Brennwald 2000). Newly synthesized secretory proteins are usually transported from one compartment of the organelle to another membrane through vesicles (Gurkan et al. 2005). They are transported to the endoplasmic reticulum at first, then transported to the plasma membrane through the Golgi apparatus, and some are delivered to the lysosome. In general, *Rab* proteins were involved in various cellular physiological functions of vesicular transport, such as cell polarity, cytokinesis, cell plate formation and so on (Barr 2009). Therefore, vesicular transport includes four steps at least. Vesicles budded from the donor membrane, moved to the receptor, anchored in the membrane receptor, and fused with membrane receptors. These processes all need the active involvement of *Rab* proteins (Tuvim et al. 2001; Yang 2002).

Up to now, the identification of *Rab* protein gene family in *Arabidopsis* (Rutherford and Moore 2002) has been very clear. But the related gene family research has not been reported in cotton. As is known to all, *Rab* gene family is a vital family in plant (Hill and Sylvester 2007). As one of the most important families in cotton, the *Rab* gene family plays a key role in the process of fiber development and biotic and abiotic stresses. With the great progress of genome sequence information in four different cotton species (*G. raimondii*, *G. arboreum*, *G. hirsutum* acc. TM-1 and *G. barbadense* acc. 3-79) (Wang et al. 2012; Li et al. 2014; Zhang et al. 2015; Yuan et al. 2015), we tried to mine the important genes related to fiber development and biotic and abiotic stresses in the whole genomic level for their functional analysis.

In this study, combined with the released genome information in four sequenced cotton species, the cotton *Rab* gene family members were systematically studied. We analyzed the characteristics on the gene structures, classification, chromosomal locations, and expression patterns of *Rab* gene family members. Our studies will lay a foundation for understanding the functional roles of different *Rab* members in the polar growth and stress tolerance in cotton.

## Methods

### Identification on *Rab* genes

Using the known *Rab* amino acid sequences (Accessions NP_187823.1 and NP_199607.1), we searched the seed file of *Rab* structure in the Pfam database (http://www.phytozome.net/) website. The number of seed file is PF00071. After that, the seed file was used to further retrieve the whole genome database of *G. raimondii* (Paterson et al. 2012) by HMMER 3.0 software (Finn et al. 2011). Finally, all obtained *Rab* proteins were used the blastp program on the NCBI site to further validate the conserved domains of proteins. The gene information of the other three cotton species, *G. arboreum*, *G. hirsutum* acc. TM-1, and *G. barbadense* acc. 3-79, were downloaded from http://cgp.genomics.org.cn, http://mascotton.njau.edu.cn and http://cotton.cropdb.org, respectively.

### Chromosomal mapping and gene duplication

We used Mapchart 2.2 software to anchor the *Rab* genes to all 13 chromosomes of *G. raimondii*. The syntenic information of *G. raimondii* was downloaded from the Plant Genome Duplication Database (PGDD; http://chibba.agtec.uga.edu/duplication/). *Rabs* were mapped to the syntenic blocks for intra- and inter-genomic comparison (Xu et al. 2016a, b). The chromosome numbers were integrated with the interspecific genetic map (D1 to D13) in allotetraploid cultivated cotton species (Wang et al. 2015) and the scaffolds (Chr.1 to Chr.13) in the genomic data of *G. raimondii* (Paterson et al. 2012).

The timing of segmental duplication events can be estimated by computing mean *Ks* values for all anchor points located in the corresponding syntenic block (Paterson et al. 2012; Wang et al. 2012), and all the *Ks* values were parsed from PGDD syntenic data. Genes separated by five or fewer genes within a 100-kb region on a chromosome may have resulted from tandem duplication (Wang et al. 2010).

### Calculating *Ka* and *Ks*

Coding sequences were aligned using the PRANK codon model with the default options, and alignment gaps were deleted manually (Loytynoja and Goldman 2005). On the basis of the aligned coding sequences, the non-synonymous substitutions per non-synonymous site (*Ka*) and the synonymous substitutions per synonymous site (*Ks*) of homologous gene pairs were computed by the maximum likelihood method in Codeml from the PAML package v4.7 (Yang 2007).

### Basic bioinformatics analysis

We use ExPASy online program (http://web.expasy.org/compute_pi/) to predict molecular weight and isoelectric point of all the *Rab* genes. Then we use Softberry online website (http://www.softberry.com) for subcellular localization prediction (Zhang et al. 2016).

### Phylogenetic and exon–intron structural analysis

Through the ClustalX software (http://www-igbmc.u-strasbg.fr/BioInfo/), all amino acid sequences of *Rab* proteins obtained were used for alignment analysis (Thompson et al. 1994). Then the results were saved with the MSF format. Later, the results were edited with the Genedoc software (http://www.psc.edu/biomed/genedoc/). We used MEGA5.0 software (http://www.megasoftware.net/index.html) to construct the phylogenetic tree of *Rab* proteins in cotton. Checking parameter Bootstrap was set to 1000 with NJ method for operation (Saitou and Nei 1987). We compared the cDNA of *Rabs* in cotton with DNA sequence and analyzed the gene structure by using online site (http://wheat.pw.usda.gov/piece/GSDraw.php).

### RNA-seq data analysis

The RNA-seq data for *Rab*s expression profile analysis was derived from TM-1 transcriptome data of Zhang et al. (2015). The data used for spatial–temporal expression analysis mainly includes the expression data of tissues and organs at different stages of growth and development.

Using RNA-seq data in the different tissues of TM-1, we filtered out the reads of multiple loci mapping, and only gene-specific read counts with unique mapping were remained to calculate the gene FPKM value, where FPKM referred to fragments per kilobase of exon model per million mapped reads with Cufflinks software (http://cufflinks.cbcb.umd.edu/). Analysis method of data model expression as follows: if the value of log_2_ FPKM is more than 1, we consider it to be expressed and if the value of log_2_ FPKM is less than or equal to 1, we consider it not to be expressed (Xu et al. 2016a, b).

## Results

### Genome-wide identification of the *Rab* gene family in *Gossypium*

Using the four sequenced cotton species (*G. raimondii*, *G. hirsutum* acc. TM-1, *G. barbadense* acc. 3-79 and *G. arboreum*), genome-wide identification of the *Rab* gene family were carried out in *Gossypium*. With several known amino acid sequences of the *Rab* gene family members, seed file (PF00071) about the *Rab* domain was queried on the website of Pfam data base. Then the protein databases of *Gossypium* were searched using HMMER 3.0 software. At last, the *Rab* domain was confirmed by a blastp program, and 87 *GrRabs*, 169 *GhRabs*, 136 *GbRabs* and 80 *GaRabs* were obtained from the four cotton species, respectively (Additional file 1).

In allotetraploid cotton *G. hirsutum* acc. TM-1, the number of *Rab* genes is about twice as many as in diploid cotton *G. raimondii* and *G. arboreum*. By comparison, in allotetraploid cotton *G. barbadense* acc. 3-79, the number of *Rab* genes is much less than that of *G. hirsutum* acc. TM-1. This might result from different sequencing methods, assembly error in partial chromosomal regions, or different degrees of colonization during the evolutionary process of *Gossypium*, and need to be further confirmed. Especially in *RabE* group, the number of *Rab* genes in *G. barbadense* has decreased drastically, implying that *Rab* members have further differentiation of function in the group *RabE* of *G. barbadense*.

The basic bioinformatics of 87 *Rabs* in *G. raimondii* were analyzed by using various softwares and online tools (Table 1). We found that the number of amino acids about 87 *GrRabs* ranged from 200 to 258 aa, which was consistent with the previous reports that the *Rab* protein usually has about more than 200 amino acids. It was also reported that the molecular weight of *Rab* proteins is generally at 20–30 kDa. In the present study, we also found that all members follow this rule, in which the minimum *Rab* protein has a molecular weight of 21.77 kDa and the maximum 28.65 kDa.

**Table 1:** Basic bioinformatics analysis of *Rab* gene family in *G. raimondii*

Gene name	Gene location	Number of amino acid (aa)	pI	Mw (kDa)	Chromosome	Subcellular location
*RabA1a*	Gorai.002G065100.1	216	5.87	24.20	Chr02	Golgi
*RabA1b*	Gorai.009G160700.1	218	5.49	24.09	Chr09	Golgi
*RabA1c*	Gorai.009G374600.1	217	6.53	24.24	Chr09	Golgi
*RabA1d*	Gorai.010G156800.1	215	6.12	23.89	Chr10	Golgi
*RabA1e*	Gorai.010G161100.1	218	5.50	24.25	Chr10	Golgi
*RabA1f*	Gorai.010G243000.1	217	5.98	24.01	Chr10	Golgi
*RabA1* *g*	Gorai.011G031200.1	217	5.70	23.90	Chr11	Golgi
*RabA1* *h*	Gorai.011G037400.1	218	5.36	24.01	Chr11	Golgi
*RabA1i*	Gorai.013G172400.1	217	5.98	24.09	Chr13	Golgi
*RabA2a*	Gorai.005G265900.1	215	6.76	23.87	Chr05	Golgi
*RabA2b*	Gorai.003G128100.1	216	6.22	23.89	Chr03	Golgi
*RabA2c*	Gorai.012G073300.1	216	6.91	24.01	Chr12	Golgi
*RabA2d*	Gorai.009G320100.1	216	6.32	24.04	Chr09	Golgi
*RabA2e*	Gorai.001G113000.1	216	7.01	23.85	Chr01	Golgi
*RabA2f*	Gorai.004G006300.1	216	5.89	23.81	Chr04	Golgi
*RabA2* *g*	Gorai.011G271400.1	214	6.32	23.67	Chr11	Golgi
*RabA2* *h*	Gorai.008G046400.1	215	6.76	23.89	Chr08	Golgi
*RabA3a*	Gorai.001G152600.1	232	5.00	25.49	Chr01	Golgi
*RabA3b*	Gorai.007G110800.1	236	5.35	26.04	Chr07	Golgi
*RabA4a*	Gorai.002G157000.1	225	6.62	24.96	Chr02	Golgi
*RabA4b*	Gorai.003G040900.1	223	9.00	24.64	Chr03	Golgi
*RabA4c*	Gorai.004G174200.1	229	6.84	25.16	Chr04	Golgi
*RabA4d*	Gorai.006G140300.1	222	5.61	24.92	Chr06	Golgi
*RabA4e*	Gorai.007G060000.1	225	6.84	24.80	Chr07	Golgi
*RabA4f*	Gorai.007G234300.1	233	6.84	25.83	Chr07	Golgi
*RabA5a*	Gorai.005G017600.1	219	5.23	24.53	Chr05	Golgi
*RabA5b*	Gorai.005G022300.1	215	5.03	24.10	Chr05	Golgi
*RabA5c*	Gorai.010G188100.1	224	5.12	24.69	Chr10	Golgi
*RabA5d*	Gorai.001G229200.1	217	4.99	24.02	Chr01	Golgi
*RabA5e*	Gorai.011G009000.1	224	5.01	24.69	Chr11	Golgi
*RabA5f*	Gorai.011G144500.1	258	6.24	28.65	Chr11	Golgi
*RabA5* *g*	Gorai.N003900.1	217	4.83	24.29	scaffold_14	Golgi
*RabA6a*	Gorai.007G004100.1	221	5.77	25.28	Chr07	Golgi
*RabA6b*	Gorai.008G289500.1	216	5.88	24.54	Chr08	Golgi
*RabB1a*	Gorai.009G185200.1	211	6.95	23.20	Chr09	Golgi
*RabB1b*	Gorai.001G224400.1	218	6.59	23.73	Chr01	Golgi
*RabB1c*	Gorai.001G267000.1	210	6.31	23.07	Chr01	Golgi
*RabB1d*	Gorai.006G035100.1	211	6.90	23.19	Chr06	Golgi
*RabB1e*	Gorai.011G093900.1	211	6.91	23.22	Chr11	Golgi
*RabB1f*	Gorai.N000100.1	211	7.01	23.42	scaffold_14	Golgi
*RabC1a*	Gorai.005G084300.1	212	5.39	23.60	Chr05	Plasma membrane
*RabC1b*	Gorai.012G035500.1	204	7.55	22.68	Chr12	Plasma membrane
*RabC1c*	Gorai.011G119600.1	208	6.41	23.25	Chr11	Plasma membrane
*RabC2a*	Gorai.002G160900.1	212	8.47	23.33	Chr02	Plasma membrane
*RabC2b*	Gorai.009G412800.1	212	5.45	23.18	Chr09	Plasma membrane
*RabC2c*	Gorai.006G161100.1	221	6.62	24.12	Chr06	Golgi
*RabC2d*	Gorai.011G234300.1	218	8.93	24.15	Chr11	Golgi
*RabC2e*	Gorai.007G372500.1	217	5.62	23.69	Chr07	Golgi
*RabD1a*	Gorai.005G266000.1	201	5.93	22.35	Chr05	Golgi
*RabD1b*	Gorai.008G046500.1	201	5.15	22.44	Chr08	Cytoplasmic
*RabD2a*	Gorai.003G111900.1	203	7.59	22.56	Chr03	Golgi
*RabD2b*	Gorai.003G176500.1	203	5.52	22.60	Chr03	Golgi
*RabD2c*	Gorai.004G070000.1	203	5.27	22.49	Chr04	Golgi
*RabD2d*	Gorai.004G194200.1	203	5.14	22.60	Chr04	Golgi
*RabD2e*	Gorai.007G085600.1	203	5.09	22.46	Chr07	Golgi
*RabE1a*	Gorai.002G253800.1	215	7.64	23.71	Chr02	Plasma membrane
*RabE1b*	Gorai.009G101400.1	217	8.37	24.01	Chr09	Cytoplasmic
*RabE1c*	Gorai.009G424200.1	215	7.64	23.72	Chr09	Plasma membrane
*RabE1d*	Gorai.010G140100.1	215	6.61	23.76	Chr10	Cytoplasmic
*RabE1e*	Gorai.006G175600.1	216	7.64	23.80	Chr06	Plasma membrane
*RabE1f*	Gorai.011G255400.1	206	5.93	22.70	Chr11	Plasma membrane
*RabE1* *g*	Gorai.013G023100.1	214	7.64	23.64	Chr13	Plasma membrane
*RabE1* *h*	Gorai.013G265100.1	215	7.64	23.62	Chr13	Golgi
*RabF1*	Gorai.011G237500.1	213	6.6	23.28	Chr11	Golgi
*RabF2a*	Gorai.002G059500.1	225	6.44	24.92	Chr02	Golgi
*RabF2b*	Gorai.009G024300.1	200	9.34	22.32	Chr09	Cytoplasmic
*RabF2c*	Gorai.009G150100.1	200	7.71	21.77	Chr09	Golgi
*RabF2d*	Gorai.010G070200.1	203	9.54	22.80	Chr10	Cytoplasmic
*RabF2e*	Gorai.010G171600.1	228	5.00	25.35	Chr10	Golgi
*RabF2f*	Gorai.010G236900.1	200	7.74	21.81	Chr10	Golgi
*RabF2* *g*	Gorai.001G005000.1	200	9.52	22.69	Chr01	Cytoplasmic
*RabF2* *h*	Gorai.006G091700.1	203	4.99	22.55	Chr06	Golgi
*RabG2*	Gorai.008G101600.1	208	5.08	23.46	Chr08	Vacuole
*RabG3a*	Gorai.005G076500.1	213	6.30	24.57	Chr05	Vacuole
*RabG3b*	Gorai.005G242800.1	207	5.06	23.05	Chr05	Vacuole
*RabG3c*	Gorai.003G064900.1	207	5.42	23.19	Chr03	Vacuole
*RabG3d*	Gorai.009G259500.1	205	5.49	23.03	Chr09	Vacuole
*RabG3e*	Gorai.006G098100.1	207	4.80	23.27	Chr06	Vacuole
*RabG3f*	Gorai.011G074700.1	208	5.37	23.27	Chr11	Vacuole
*RabG3* *g*	Gorai.008G028300.1	206	5.09	23.01	Chr08	Vacuole
*RabG3* *h*	Gorai.013G037800.1	207	5.10	23.22	Chr13	Vacuole
*RabH1a*	Gorai.004G037900.1	208	6.38	23.18	Chr04	Golgi
*RabH1b*	Gorai.004G067600.1	208	7.70	23.20	Chr04	Golgi
*RabH1c*	Gorai.007G060700.1	208	7.67	23.09	Chr07	Golgi
*RabH1d*	Gorai.007G238500.1	208	6.38	23.18	Chr07	Golgi
*RabH1e*	Gorai.008G090100.1	207	7.67	23.17	Chr08	Golgi
*RabH1f*	Gorai.008G111500.1	208	5.94	23.10	Chr08	Golgi

### The nomenclature of the *Rab* gene family in *Gossypium*

**Fig. 1:**
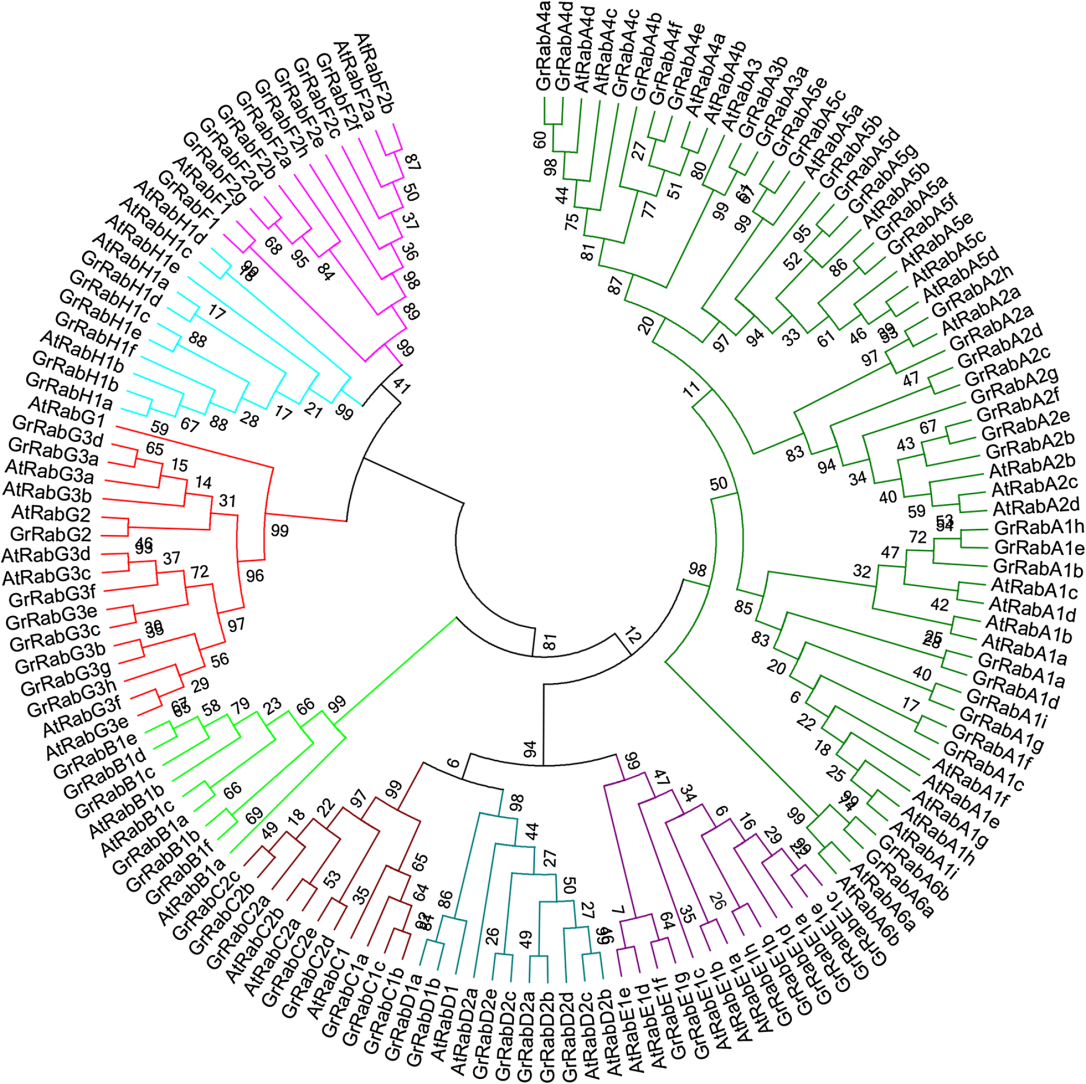
Phylogenetic relationships of *Rab* family members between *G. raimondii* and *Arabidopsis*. The multiple alignment of the conserved *Rab* family domain between *G. raimondii* and *Arabidopsis* were constructed with Clustal X (version 2.0). Phylogenetic tree was generated using the maximum likelihood method under WAG model in MEGA v5.2, and the reliability of interior branches was assessed with 1000 bootstrap resamplings

In total, 57 *Rabs* in *A. thaliana* were identified. Then they were divided into eight groups and eighteen subgroups. These groups were named as *AtRabA*, *AtRabB*, *AtRabC*, *AtRabD*, *AtRabE*, *AtRabF*, *AtRabG* and *AtRabH*, respectively. According to cluster analysis of 57 members in *A. thaliana* and 87 in *G. raimondii*, we found that 87 *GrRabs* were also divided into eight groups, and named as *GrRabA*, *GrRabB*, *GrRabC*, *GrRabD*, *GrRabE*, *GrRabF*, *GrRabG* and *GrRabH*, correspondingly. There were 17 subgroups in *G. raimondii* compared with 18 subgroups in *A. thaliana* (Fig. 1). The number of each subgroup was named by combining their chromosome order (D1 to D13) with their location on the chromosome. Based on the nomenclature of *G. raimondii*, the corresponding *Rab* orthologs in *G. arboreum*, *G. hirsutum* acc. TM-1, and *G. barbadense* acc. 3-79 were named, respectively, with the same number for orthologs as in *G. raimondii* (Additional file 1).

We counted the numbers and proportion of *Rab* genes of the eight groups between *Arabidopsis* and *G. raimondii* (Tables 2 and 3). As we can see that *RabA* is the largest group of the family and the largest proportion. The proportion of the same group was similar between *Arabidopsis* and *G. raimondii*. According to the position of each member in the phylogenetic tree, combining with the function of reported genes in the same group, we speculated that there were the similar function in the same group.

**Table 2:** The numbers of *Rab* genes of the eight groups between *Arabidopsis* and *G. raimondii*

	*RabA*	*RabB*	*RabC*	*RabD*	*RabE*	*RabF*	*RabG*	*RabH*
*Arabidopsis*	26	3	3	4	5	3	8	5
*G. raimondii*	34	6	8	7	8	9	9	6

**Table 3:** The proportion of *Rab* genes in the eight groups between *Arabidopsis* and *G. raimondii*

	*RabA* (%)	*RabB* (%)	*RabC* (%)	*RabD* (%)	*RabE* (%)	*RabF* (%)	*RabG* (%)	*RabH* (%)
*Arabidopsis*	45.61	5.26	5.26	7.02	8.77	5.26	14.04	8.77
*G. raimondii*	39.08	6.90	9.20	8.05	9.20	10.34	10.34	6.90

### The chromosome distribution of the *Rab* gene family in *G. raimondii*

**Fig. 2:**
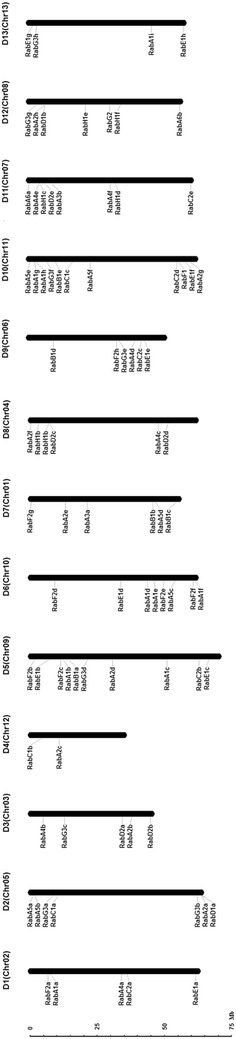
Chromosomal distribution of *Rab* genes in *G. raimondii*. The chromosome numbers were consistent with the interspecific genetic map (D1 to D13) in allotetraploid cultivated cotton species (Wang et al. 2015) and the scaffolds (Chr.1 to Chr.13) in the genomic data of *G. raimondii* (Paterson et al. 2012). The nomenclature of *Rabs* was based on *Arabidopsis* nomenclature and the order of the chromosomes in *G. raimondii*

To elucidate the chromosomal distribution of these *Rab* genes, we integrated 13 scaffolds of the *G. raimondii* genome (named as Chr. 1 to Chr. 13) with the reported high-density interspecific genetic map of allotetraploid cultivated cotton species (Wang et al. 2015). Using Mapchart 2.2 software and the genomic localization information, we mapped the distribution of 87 *GrRab* genes on 13 chromosomes of *G. raimondii* (Fig. 2). 85 *GrRab* genes were tagged on the corresponding physical location of all the 13 chromosomes except for two genes, indicating that the scaffolds involved in the two genes had not been integrated in the chromosome in the present draft genome. The chromosomal distribution patterns of these *GrRabs* were uneven. Chr.11 (D10) contained the most *Rabs* (11 genes), while Chr.12 (D4) contained the fewest (two genes).

### *Rab* gene family expansion in *G. raimondii*

To investigate the *Rab* gene family expansion pattern in cotton, we download the syntenic data of *G. raimondii* from the Plant Genome Duplication Database (PGDD). We identified the number of *Rab* genes formed in whole genome duplication (WGD) and tandem duplication in cotton. Among the 87 *GrRabs* in *G. raimondii*, we identified 60 pairs of *GrRabs* formed in whole genome duplication (WGD) (Additional file 2). We also analyzed the adjacent genes to investigate whether tandem duplication had taken place. Four of the 87 *GrRabs* showed tandem duplication. They were *GrRabA2a* vs *GrRabD1a* and *GrRabA2h* vs *GrRabD1b* respectively.

In 60 pairs of whole genome duplication gene pairs (Additional file 2), we obtained the *Ks* values of 58 pairs of whole genome duplication gene pairs. Of them, the *Ks* values of eight pairs of genes are greater than 1. They were *GrRabB1b* vs *GrRabB1a*, *GrRabC2c* vs *GrRabC2d*, *GrRabE1a* vs *GrRabE1g*, *GrRabE1e* vs *GrRabE1b*, *GrRabE1b* vs *GrRabE1f*, *GrRabE1b* vs *GrRabE1g*, *GrRabE1e* vs *GrRabE1f* and *GrRabE1g* vs *GrRabE1h*, respectively. It was found that the majority of the gene pairs come from *GrRabE* group. These gene pairs may be derived from the ancient event. For the remaining 50 gene pairs, the *Ks* values ranged from 0.331 to 0.8772, implying that these gene pairs originated from the *Gossypium* lineage WGD events. Among all the gene pairs, the *Ka*/*Ks* values were less than 1, which indicated the results of the purification selection, and help maintain the conservation of the duplicated genes in structure and function.

### Exon–intron structural analysis and subcellular localization prediction

**Fig. 3:**
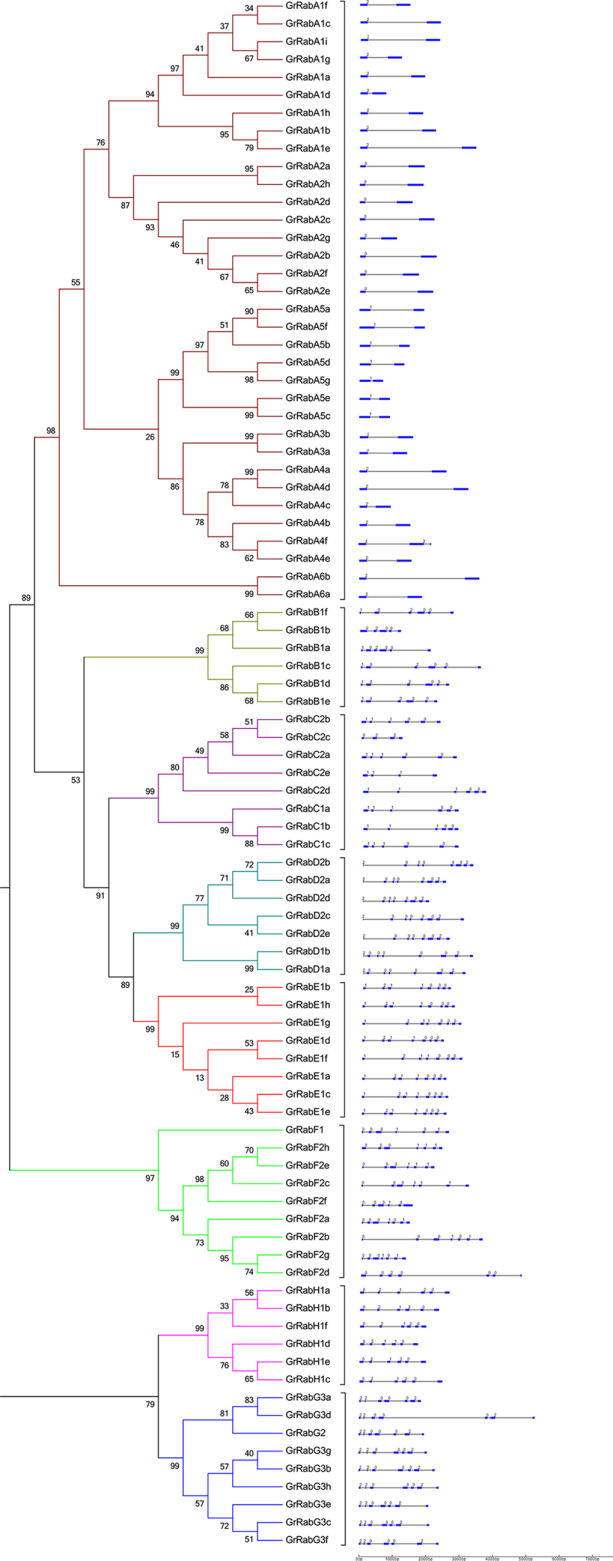
Phylogenetic relationships and gene structures of *Rabs* in cotton. The multiple alignment of the conserved *Rab* family domain were constructed with Clustal X (version 2.0). Phylogenetic tree was generated using the maximum likelihood method under WAG model in MEGA v5.2, and the reliability of interior branches was assessed with 1000 bootstrap resamplings. The gene structures were drawn using the online tool GSDS. Introns and exons were represented by *black lines* and *blue boxes*, respectively, and numbers at the exon–intron joints were intron phases

In order to better understand the genetic structure of 87 *Rab* gene family members in *G. raimondii*, we obtained the exon and intron structure distribution of each gene by combining their genome sequence with CDS sequence (Fig. 3) using the online website (http://wheat.pw.usda.gov/piece/GSDraw.php). The exon and intron gene structure analysis showed that all 87 *Rab*s had different introns. It is worth mentioning that the number of introns is different, but the genes clustered together have a very similar distribution of exons and introns. For 87 *Rab* protein gene family members, we compared the numbers of introns in each class. In all of 34 *RabA* family members, we found that there is only one intron among the 33 *Rabs* with one exception. Among 6 *RabB* family members, there are 5 introns. Then, we found that there are 5 introns among the most of *RabC* family members. All *Rab*s in *RabD* and *RabE* family contain 7 introns. Among 9 *RabF* family members, there are 8 *Rabs* containing 6 introns. Finally, all *Rab*s contained 6 introns in *RabG* family and 5 introns in *RabH* family. It can be concluded that intron numbers of most *Rab*s in the same group are basically the same, only a few genes exception.

Using the Softberry online software for subcellular localization prediction, we found that 87 *Rabs* mainly concentrated in organelles with membrane structure and cell membrane (Table 1). We further found that all members of *RabA*, *RabB* and *RabH* are located in the Golgi, *RabGs* are located in the vacuole, *RabCs* are distributed in the cell membrane or Golgi, *RabDs* and *RabFs* are distributed in the cytoplasmic or Golgi, *RabEs* are distributed in the cell membrane or cytoplasmic.

Taken together, the genes clustered together have similar gene structure and subcellular localization, implying *Rab* genes in the same class generally have similar functions.

### Expression pattern analysis

**Fig. 4:**
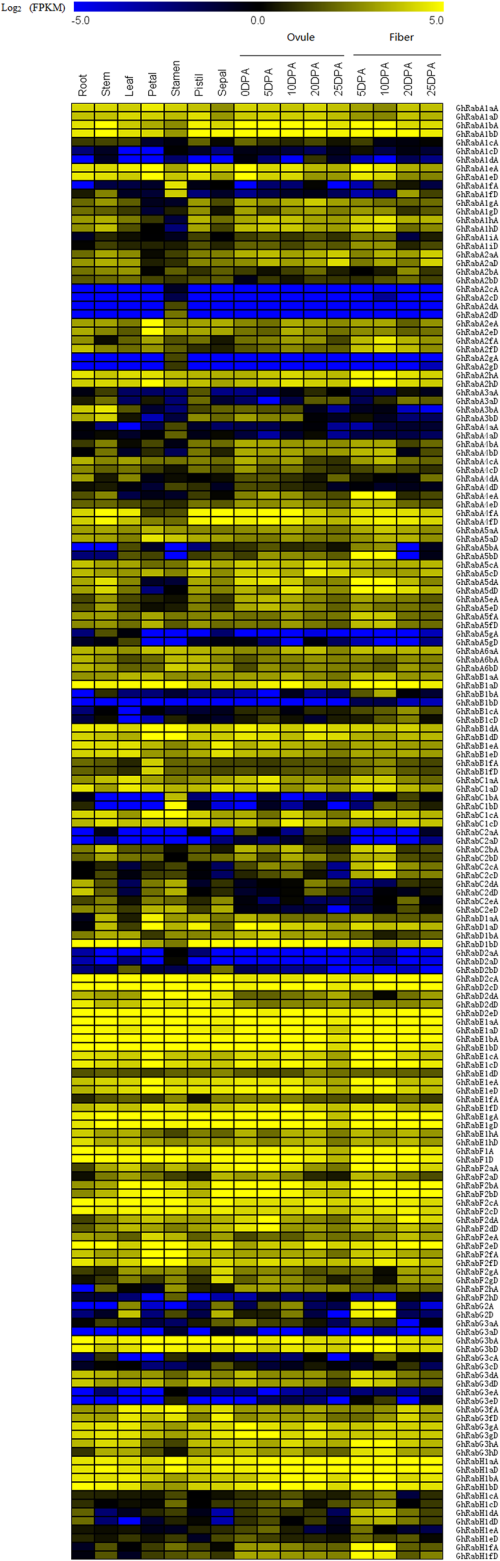
Expression profiles of *Rab* genes in cotton. The RNA-seq relative expression data of 16 tissues was used to re-construct expression patterns of 169 *Rab* genes in *G. hirsutum* acc. TM-1. Analysis method of data model expression as follows: if the value of log_2_ FPKM is more than 1, we consider it to be expressed and if the value of log_2_ FPKM is less than or equal to 1, we consider it not to be expressed

The analysis of gene expression patterns can provide powerful clues and help for prediction of gene function. We found that cotton *Rabs* are expressed in all tissues of plant and are regulated temporally and spatially depending on developmental stage and environmental conditions (Additional files 3, 4). Among the 169 *GhRabs* (Fig. 4), there were 161 genes with log_2_ FPKM > 1 in at least one of the 16 investigated organs and developmental stages, and these 161 genes were used to gauge the relative expression of each *Rab* gene. It indicates that most *Rab* family members play a certain role in different growth stages of cotton. The remaining 8 *GhRabs* may be pseudogenes or only expressed under special environmental conditions. About half of the genes (86 *GhRabs*) were expressed widely in vegetative organs, flower organs and reproductive organs. It shows that they are constitutively expressed and participate in the whole growth and development process of cotton. Approximately 10.65% (18 *GhRabs*) of the genes were expressed preferentially in cotton fibers, and most of them were highly expressed at the elongation stage of fiber development, indicating that these genes may be involved in regulating the growth and development of cotton fiber. A few genes are dominantly expressed in floral organs, suggesting that they may play a role in pollen formation and development. A small number of genes were preferentially expressed in the root, stem and leaf, suggesting that they might be involved in the process of plant stress resistance. We also observed the significant different expression patterns among each group of *Rab* family.

## Discussion

It is reported that *Rab* genes played the various roles in all kinds of plants. Through cotton genome sequence information, we tried to analyze the basic biological information, gene structure, genome distribution, and expression characteristics of *Rab* gene family members. We hope to find some important genes related to stress response and fiber development in the whole genomic level, and help to utilize them to improve the quality and yield of cotton.

### Consistency on classification and structure of 87 *GrRabs*

In *Arabidopsis*, a total of 57 *Rab* members are divided into eight groups. Similarly, 87 *Rabs* in *G. raimondii* are also divided into eight groups. We found that although the length of 87 *Rabs* and the numbers of introns are different, it is worth mentioning that the genes clustering together have a very similar distribution of exon and intron structure and subcellular localization. The intron numbers of 87 *GrRabs* in the same group are basically the same. It is believed that the genes clustered together have similar gene structure and the genes in the same class have similar functions.

Previous studies on many *Rab* proteins in *Arabidopsis* have been reported in detail. However, the study of Rab proteins in cotton is few. According to the function of *Rab* proteins in *Arabidopsis* (Rutherford and Moore 2002), we can infer the possible function roles of *Rab* proteins in cotton by clustering *Rab* members in *Arabidopsis* and *G. raimondii*. It has a guiding role for further study of gene function by genomic-wide comparative analysis among different plant species.

### The functional diversity of *Rab* proteins

We used the transcriptome sequencing results of upland cotton genetic standard line TM-1 and carried out expression pattern analysis on 169 *GhRabs*. We found that 161 of 169 *GhRabs* have a certain amount of expression level in at least one of the 16 investigated organs and developmental stages. We speculate that the vast majority of *Rab* genes have certain function in cotton growth and development. About half of the genes (86 *GhRabs*) were expressed in all kinds of tissues and organs. In addition, some genes specifically expressed in certain tissues and organs, implying that *Rab* genes have diversity on the function. Referring the reports of *Rab*s function and their expression profile in cotton, cotton *Rabs* may involve in the biotic and abiotic stress response, pollen germination and pollen tube elongation, seed germination and fiber development.

In the previous study, it is reported that many *Rab* proteins are involved in important functions in a lot of plants. Among them, the hottest spot of current research is that *Rab* protein was involved in cell polarity growth. In the plant growth process, polar growth process is very important. The polarity growth process in plant includes elongation of pollen tubes (Peng et al. 2011) and root hairs (Preuss et al. 2006; Blanco et al. 2009). This process requires vesicle trafficking and microtubule actin proteins. *Rab* protein is an important molecular switch in the cell, which controls the transport of vesicles in various ways, so as to promote the establishment and maintenance of apical polarity (Cole and Fowler 2006; Samaj et al. 2006).

In *Arabidopsis*, root hairs and pollen tubes elongate through the polar growth (Campanoni and Blatt 2007). Although they have different origins and functions, these cells promote cell elongation by Ca^2+^ enrichment at the cell tip and actin cytoskeleton regulation (Li and Yang 1999; Hepler et al. 2001; Samaj et al. 2005; Cole and Fowler 2006). The overexpression of *Rab11b* inhibits the elongation of pollen tube and influence the direction of pollen tube growth (de Graaf et al. 2005). *NtRab2*, which is overexpressed in tobacco, also inhibits the growth of pollen tube (Cheung et al. 2002). In *Arabidopsis*, *RabA4d* is necessary for the proper regulation of pollen tube growth. The loss of *RabA4d* leads to the destruction of polar growth and changes in the structure of the cell wall. These results indicate that *RabA4d* plays an important role in regulating the growth of pollen tube (Szumlanski and Nielsen 2009). Enhanced yellow fluorescent protein *RabA4b*-EYFP specifically located in the tip of the root hair cells of *Arabidopsis*. In root hair defective mutants, yellow fluorescence signal localization of *RabA4b*-EYFP in hair cells no longer exist. As a result, *RabA4b* potentially regulates membrane transport in plant cells through the involvement of cell wall components in the secretory process (Preuss and Nielsen 2004). *AtRab2* was specifically expressed in newly germinated seedlings and pollen tubes. The GUS connected to the promoter of the gene was clearly detected in mitosis of pollen tubes. After that, its expression level has been increased, indicating that *AtRab2* plays an important role in the growth and development of pollen tubes (Moore et al. 1997). *Rgp1* isolated from rice is a gene homologous to yeast *Ypt3*. After overexpression of this gene in tobacco plants, the plants exhibited dwarf and the abnormal development of floral organs. Of them, the transgenic plants were more than 6 times higher in the content of endogenous hormones than that in the wild plants. These results suggest that *Rgp1* may be involved in cytokinin signaling pathways (Kamada et al. 1992; Sano et al. 1994). Thus, the *Rab* protein is essential for root hairs and pollen tube elongation of plant. Cotton fiber cells, root hairs and pollen tubes are typical tissues for studying polar growth of plants. We hypothesized that *Rab* proteins would be involved in the fiber polarity elongation process and plant growth process in cotton.

*Rab* proteins not only play an important role in plant growth and development, but also play an important role in biotic and abiotic stress responses. When the relative water content of leaves decreased, the expression of *Rab2* increased. Further, the expression level of *Rab2* was increased when exogenous ABA was applied to the restored grass plants. The results show that *Rab2* plays an important role in drought stress (O’Mahony and Oliver 1999). Under a variety of environments (chilling, salinity, drying and ABA) induction, the expression level of *OsRab7* significantly altered. Through the transformation of *Arabidopsis* protoplasts, the *OsRab7* fusion protein of GFP was localized in the vacuole. It is suggested that *OsRab7* is transported to the vacuole through the vesicle transport in plant cells. The inducible expression of *OsRab7* suggests that it is involved in the response of these stresses, which suggests that *Rab* is related to abiotic stresses (Nahm et al. 2003). Overexpression of *AtRabG3e* in *Arabidopsis* could enhance the resistance of plants to salt and osmotic stress. The enhancement of plant resistance was related to the acceleration of endocytosis and the increase of sodium ion in vacuole (Mazel et al. 2004). In addition, when *AtRabG3b* was silenced, there was no difference in the silenced plants compared with the no transgenic plants. However, when *AtRabG3b* was overexpressed, it showed hypersensitive cell death on pathogenic fungi and mycotoxins. In addition, it also accelerated leaf senescence. These results suggest that *AtRabG3b* is involved in the regulation of cell apoptosis and the regulation of pathogen response during plant senescence (Kwon et al. 2009). Transgenic tobacco plants overexpressing *PgRab7* also showed resistance to drought and salt stress, which indicated that *PgRab7* was involved in plant stress response (Agarwal et al. 2008). *OsRab11* is widely expressed in various tissues and organs of plants, which is induced by jasmonic acid (JA). Overexpression of *OsRab11* in transgenic plants enhanced the resistance to pathogens by affecting the expression of genes involved in the metabolic pathway of jasmonic acid. Therefore, *OsRab11* is necessary in the jasmonic acid mediated signaling pathway (Hong et al. 2013). Based on above, we hypothesized that *Rab* genes in cotton play an important role in biotic and abiotic stress responses.

## Conclusions

*Rab* proteins play an important role in plant growth and development, as well as in biotic and abiotic stress responses. In this study, we individually identified 87, 169, 136, 80 *Rabs* in the four sequenced cotton species. These *Rabs* are divided into eight groups. In each group, their intron numbers and subcellular localization are basically the same. Further, 60 pairs of segmental duplication due to whole genome duplication and two pairs of tandem duplication were detected, respectively. Expression patterns analysis indicated that most *Rab* family members play a certain role in different tissues/organs and different growth stages of cotton, implying their potential function in the polar growth and stress tolerance.

## Additional files

**Additional file 1.** List of Rab genes in *G. raimondii*, *G. hirsutum*, *G. barbadense* and *G. arboreum*, respectively.

**Additional file 2.** Ka, Ks and Ka/Ks values of gene pairs in syntenic blocks.

**Additional file 3.** The transcriptome data (FPKM) of Rabs in distinct tissues.

**Additional file 4.** The transcriptome data (log_2_FPKM) of *Rabs* in distinct tissues.

## Abbreviations

*Rab*: ras-like in rat brain
PGDD: plant genome duplication database
WGD: whole genome duplication
FPKM: fragments per kilobase of exon model per million mapped reads
*Ka*: nonsynonymous substitutions per nonsynonymous site
*Ks*: synonymous substitutions per synonymous site

## References

[CR1] Agarwal PK, Agarwal P, Jain P, Jha B, Reddy MK, Sopory SK (2008). Constitutive overexpression of a stress-inducible small GTP-binding protein *Pgrab7* from *Pennisetum glaucum* enhances abiotic stress tolerance in transgenic tobacco. Plant Cell Rep.

[CR2] Agarwal P, Reddy MK, Sopory SK, Agarwal PK (2009). Plant *Rabs*: characterization, functional diversity, and role in stress tolerance. Plant Mol Biol Rep.

[CR3] Barr FA (2009). *Rab* GTPase function in Golgi trafficking. Semin Cell Dev Biol.

[CR4] Blanco FA, Peltzer Meschini E, Zanetti ME, Aguilar OM (2009). A small GTPase of the *Rab* family is required for root hair formation and preinfection stages of the common bean-Rhizobium symbiotic association. Plant Cell.

[CR5] Brennwald P (2000). Reversal of fortune: do *Rab* GTPases act on the target membrane?. J Cell Biol.

[CR6] Brighouse A, Dacks JB, Field MC (2010). *Rab* protein evolution and the history of the eukaryotic endomembrane system. Cell Mol Life Sci.

[CR7] Campanoni P, Blatt MR (2007). Membrane trafficking and polar growth in root hairs and pollen tubes. J Exp Bot.

[CR8] Cheung AY, Christine Y-HC, Glaven RH, Graaf BHJD, Luis V, Hepler PK, Wu HM (2002). *Rab2* GTPase regulates vesicle trafficking between the endoplasmic reticulum and the Golgi bodies and is important to pollen tube growth. Plant Cell.

[CR9] Cole RA, Fowler JE (2006). Polarized growth: maintaining focus on the tip. Curr Opin Plant Biol.

[CR10] de Graaf BH, Cheung AY, Andreyeva T, Levasseur K, Kieliszewski M, Wu HM (2005). *Rab11* GTPase-regulated membrane trafficking is crucial for tip-focused pollen tube growth in tobacco. Plant Cell.

[CR11] Finn RD, Clements J, Eddy SR (2011). HMMER web server: interactive sequence similarity searching. Nucleic Acids Res.

[CR12] Gallwitz D, Donath C, Sande C (1983). A yeast gene encoding a protein homologous to the human c-has/bas proto-oncogene product. Nature.

[CR13] Gurkan C, Lapp H, Alory C, Su AI, Hogenesch JB, Balch WE (2005). Large-scale profiling of *Rab* GTPase trafficking networks: the membrome. Mol Biol Cell.

[CR14] Hepler PK, Vidali L, Cheung AY (2001). Polarized cell growth in higher plants. Annu Rev Cell Dev Biol.

[CR15] Hill D, Sylvester A (2007). Diversification of the *Rab* guanosine triphosphatase family in dicots and monocots. J Integr Plant Biol.

[CR16] Hong MJ, Yun ML, Son YS, Im CH, Yi YB, Rim YG, Bahk JD, Heo JB (2013). Rice *Rab11* is required for JA-mediated defense signaling. Biochem Biophys Res Commun.

[CR17] Kamada I, Yamauchi S, Youssefian S, Sano H (1992). Transgenic tobacco plants expressing rgpl, a gene encoding ras-related GTPbinding protein from rice, show distinct morphological characterization. Plant J.

[CR18] Kwon SI, Hong JC, Bae K, Jung JH, Jin HC, Park OK (2009). Role of an *Arabidopsis*, *Rab* GTPase *RabG3b* in pathogen response and leaf senescence. J Plant Biol.

[CR19] Li H, Yang Z (1999). Control of pollen tube tip growth by a *Rop* GTPase-dependent pathway that leads to tip-localized calcium influx. Plant Cell.

[CR20] Li F, Fan G, Wang K, Sun F, Yuan Y, Song G, Li Q, Ma Z, Lu C, Zou C, Chen W, Liang X, Shang H, Liu W, Shi C, Xiao G, Gou C, Ye W, Xu X, Zhang X, Wei H, Li Z, Zhang G, Wang J, Liu K, Kohel RJ, Percy RG, Yu JZ, Zhu YX, Wang J, Yu S (2014). Genome sequence of the cultivated cotton *Gossypium arboretum*. Nat Genet.

[CR21] Loytynoja A, Goldman N (2005). An algorithm for progressive multiple alignment of sequences with insertions. Proc Natl Acad Sci USA.

[CR22] Martinez O, Goud B (1998). *Rab* proteins. Biochim Biophys Acta.

[CR23] Mazel A, Leshem Y, Tiwari BS, Levine A (2004). Induction of salt and osmotic stress tolerance by overexpression of an intracellular vesicle trafficking protein *AtRab7* (*AtRabG3e*). Plant Physiol.

[CR24] Moore I, Diefenthal T, Zarsky V, Schell J, Palme K (1997). A homolog of the mammalian GTPase *Rab2* is present in *Arabidopsis* and is expressed predominantly in pollen grains and seedlings. Proc Natl Acad Sci USA.

[CR25] Nahm MY, Kim SW, Yun D, Lee SY, Cho MJ, Bahk JD (2003). Molecular and biochemical analyses of *OsRab7*, a rice *Rab7* homolog. Plant Cell Physiol.

[CR26] Novick P, Zerial M (1997). The diversity of *Rab* proteins in vesicle transport. Curr Opin Cell Biol.

[CR27] O’Mahony PJ, Oliver MJ (1999). Characterization of adesiccation-responsive small GTP-binding protein (*Rab2*) from thedesiccation-tolerant grass *Sporobolus stapfianus*. Plant Mol Biol.

[CR28] Paterson AH, Wendel JF, Gundlach H, Guo H, Jenkins J, Jin D, Llewellyn D, Showmaker KC, Shu S, Udall J, Yoo MJ, Byers R, Chen W, Doron-Faigenboim A, Duke MV, Gong L, Grimwood J, Grover C, Grupp K, Hu G, Lee TH, Li J, Lin L, Liu T, Marler BS, Page JT, Roberts AW, Romanel E, Sanders WS, Szadkowski E, Tan X, Tang H, Xu C, Wang J, Wang Z, Zhang D, Zhang L, Ashrafi H, Bedon F, Bowers JE, Brubaker CL, Chee PW, Das S, Gingle AR, Haigler CH, Harker D, Hoffmann LV, Hovav R, Jones DC, Lemke C, Mansoor S, ur Rahman M, Rainville LN, Rambani A, Reddy UK, Rong JK, Saranga Y, Scheffler BE, Scheffler JA, Stelly DM, Triplett BA, Van Deynze A, Vaslin MF, Waghmare VN, Walford SA, Wright RJ, Zaki EA, Zhang T, Dennis ES, Mayer KF, Peterson DG, Rokhsar DS, Wang X, Schmutz J (2012). Repeated polyploidization of *Gossypium* genomes and the evolution of spinnable cotton fibres. Nature.

[CR29] Peng J, Ilarslan H, Wurtele ES, Bassham DC (2011). *AtRabD2b* and *AtRabD2c* have overlapping functions in pollen development and pollen tube growth. BMC Plant Biol.

[CR30] Pereira-Leal JB, Seabra MC (2001). Evolution of the *Rab* family of small GTP-binding proteins. J Mol Biol.

[CR31] Preuss ML, Nielsen E (2004). The *Arabidopsis Rab* GTPase *RabA4b* localizes to the tips of growing root hair cells. Plant Cell.

[CR32] Preuss ML, Schmitz AJ, Thole JM, Bonner HK, Otegui MS, Nielsen E (2006). A role for the *RabA4b* effector protein PI-4K1 in polarized expansion of root hair cells in *Arabidopsis thaliana*. J Cell Biol.

[CR33] Rutherford S, Moore I (2002). The *Arabidopsis*, Rab GTPase family: another enigma variation. Curr Opin Plant Biol.

[CR34] Saitou N, Nei M (1987). The neighbor-joining method: a new method for reconstructing phylogenetic trees. Mol Biol Evol.

[CR35] Salminen A, Novick PJ (1987). A ras-like protein is required for a post-Golgi event in yeast secretion. Cell.

[CR36] Samaj J, Read ND, Volkmann D, Menzel D, Baluska F (2005). The endocytic network in plants. Trends Cell Biol.

[CR37] Samaj J, Muller J, Beck M, Böhm N, Menzel D (2006). Vesicular trafficking, cytoskeleton and signalling in root hairs and pollen tubes. Trends Plant Sci.

[CR38] Sano H, Seo S, Orudgev E, Youssefian S, Ishizuka K (1994). Expression of the gene for a small GTP binding protein in transgenic tobacco elevates endogenous cytokinin levels, abnormally induces salicylic acid in response towounding, and increases resistance to tobacco mosaic virus infection. Proc Natl Acad Sci USA.

[CR39] Stenmark H, Olkkonen VM (2001). The *Rab* GTPase family. Genome Biol.

[CR40] Szumlanski AL, Nielsen E (2009). The *Rab* GTPase *RabA4d* regulates pollen tube tip growth in *Arabidopsis thaliana*. Plant Cell.

[CR41] Takai Y, Sasaki T, Matozaki T (2001). Small GTP-binding proteins. Physiol Rev.

[CR42] Thompson JD, Higgins DG, Gibson TJ (1994). CLUSTAL W, (improving the sensitivity of progressive multiple sequence alignment through sequence weighting, position-specific gap penalties and weight matrix choice). Nucleic Acids Res.

[CR43] Tuvim MJ, Adachi R, Hoffenberg S, Dickey BF (2001). Traffic control: *Rab* GTPases and the regulation of interorganellar transport. News Physiol Sci.

[CR44] Wang L, Guo K, Li Y, Tu Y, Hu H, Wang B, Cui X, Peng L (2010). Expression profiling and integrative analysis of the CESA/CSL, superfamily in rice. BMC Plant Biol.

[CR45] Wang K, Wang Z, Li F, Ye W, Wang J, Song G, Yue Z, Cong L, Shang H, Zhu S, Zou C, Li Q, Yuan Y, Lu C, Wei H, Gou C, Zheng Z, Yin Y, Zhang X, Liu K, Wang B, Song C, Shi N, Kohel RJ, Percy RG, Yu JZ, Zhu YX, Wang J, Yu S (2012). The draft genome of a diploid cotton *Gossypium raimondii*. Nat Genet.

[CR46] Wang S, Chen JD, Zhang WP, Hu Y, Chang LJ, Fang L, Wang Q, Lv FN, Wu HT, Si ZF, Chen SQ, Cai CP, Zhu XF, Zhou BL, Guo WZ, Zhang TZ (2015). Sequence-based ultra-dense genetic and physical maps reveal structural variations of allopolyploid cotton genomes. Genome Biol.

[CR47] Xu J, Xu X, Tian L, Wang G, Zhang X, Wang X, Guo W (2016). Discovery and identification of candidate genes from the chitinase gene family for *Verticillium dahliae* resistance in cotton. Sci Rep.

[CR48] Xu X, Feng Y, Fang S, Xu J, Wang X, Guo W (2016). Genome-wide characterization of the β-1,3-glucanase gene family in *Gossypium* by comparative analysis. Sci Rep.

[CR49] Yang Z (2002). Small GTPases: versatile signaling switches in plants. Plant Cell.

[CR50] Yang Z (2007). PAML 4: phylogenetic analysis by maximum likelihood. Mol Biol Evol.

[CR51] Yuan D, Tang Z, Wang M, Gao W, Tu L, Jin X, Chen L, He Y, Zhang L, Zhu L, Li Y, Liang Q, Lin Z, Yang X, Liu N, Jin S, Lei Y, Ding Y, Li G, Ruan X, Ruan Y, Zhang X (2015). The genome sequence of Sea-Island cotton (*Gossypium barbadense*) provides insights into the allopolyploidization and development of superior spinnable fibres. Sci Rep.

[CR52] Zhang T, Hu Y, Jiang W, Fang L, Guan X, Chen J, Zhang J, Saski CA, Scheffler BE, Stelly DM, Hulse-Kemp AM, Wan Q, Liu B, Liu C, Wang S, Pan M, Wang Y, Wang D, Ye W, Chang L, Zhang W, Song Q, Kirkbride RC, Chen X, Dennis E, Llewellyn DJ, Peterson DG, Thaxton P, Jones DC, Wang Q, Xu X, Zhang H, Wu H, Zhou L, Mei G, Chen S, Tian Y, Xiang D, Li X, Ding J, Zuo Q, Tao L, Liu Y, Li J, Lin Y, Hui Y, Cao Z, Cai C, Zhu X, Jiang Z, Zhou B, Guo W, Li R, Chen ZJ (2015). Sequencing of allotetraploid cotton (*Gossypium hirsutum* L. acc. TM-1) provides a resource for fiber improvement. Nat Biotechnol.

[CR53] Zhang X, Xu X, Yu Y, Chen C, Wang J, Cai C, Guo W (2016). Integration analysis of MKK and MAPK family members highlights potential MAPK signaling modules in cotton. Sci Rep.

